# Third party drug checking: accessing harm reduction services on the behalf of others

**DOI:** 10.1186/s12954-021-00545-w

**Published:** 2021-09-17

**Authors:** Ashley Larnder, Piotr Burek, Bruce Wallace, Dennis K. Hore

**Affiliations:** 1 Department of Chemistry, University of Victoria, Victoria, V8W 3V6 Canada; 2 Department of Computer Science, University of Victoria, Victoria, V8W 3P6 Canada; 3 School of Social Work, University of Victoria, Victoria, V8W 2Y2 Canada; 4 Canadian Institute for Substance Use Research, University of Victoria, Victoria, V8W 2Y2 Canada

**Keywords:** People who use drugs, Harm reduction, Drug checking, Overdose, Third party, Community-based

## Abstract

**Background:**

Drug checking uses chemical analytical technologies to analyze drugs from the unregulated market to reduce substance use-related risks. We aim to examine the frequency of third party use of a community drug checking service to explore the potential for harm reduction to extend beyond the individual into the community, increase service accessibility, and to contribute to upstream interventions in the supply.

**Methods:**

Over 31 months, data were collected from a point-of-care drug checking service operated in Victoria, Canada. Through the implementation of survey questions at the intake of the service, data were collected about whether the drug check was for the individual, to sell, and/or for others.

**Results:**

Just over half (52%) of service users were checking for reasons that extended beyond individual use. When checking for others, friends were the most common response, representing 52% of responses, and outreach/support workers checking for others was the second most at 32%. Twelve percent of service users reported checking to sell or for a supplier.

**Conclusions:**

Third party checking is a frequent, and important aspect of drug checking services, which through facilitating community engagement and increasing accessibility, has expanded the reach of interventions beyond individuals to reduce risks within the unregulated market. Therefore, drug checking as an overdose response should be responsive and accessible for those using the service on the behalf of others.

## Introduction

Increasingly, drug checking services are being explored as an intervention in the ongoing overdose epidemic [1–3]. Drug checking aims to provide information on the composition of illicit drugs from the unregulated market to reduce substance use related risks. Services associated with dance and festival communities have been identified as an important facilitator of individual harm reduction practices [4–6]. Though often evaluated as an individual behavioral interventions alone, drug checking is also being explored by some for its potential as an intervention within the broader community and drug market [7–9]. Wallace et al. [9] highlight the potential of drug checking to act as a supply intervention, showcasing how the propensity to evaluate drug checking on individual abstinence-based outcomes can detract from its potential role as an upstream community intervention that has the capability to reduce stigma and improve population health.

Third party drug checking, which we define as accessing drug checking on someone else’s behalf or in a combination for self and others, has the opportunity to expand service accessibility and to reduce risks within a larger group beyond the individual level interaction. In this brief report, we showcase results from a community drug checking service to examine the frequency of third party access and to explore the reach of this practice to extend drug checking benefits beyond the individual.

## Methods

Data were collected from a drug checking service in Victoria, Canada between November 10, 2018, and June 29, 2021, with ethical approval from the Health Research Ethics Board at the Island Health Authority (J2018-069). The service implements chemical analytical technologies, including fentanyl and benzodiazepine immunoassay test strips, Raman spectroscopy, infrared absorption spectroscopy, and paper spray–mass spectrometry [3]. Over time, the location of the service changed, but has always been established alongside community harm reduction sites and services, with origins in overdose prevention sites. The current site is a store-front location within a building operated by the local drug user organization and alongside their related services. Service is currently operated from 12-7 pm, Monday through Friday. This is the only drug checking program available in-person in Victoria, Canada.

At the point of intake, service users were asked preliminary survey questions about the sample to guide the drug check. This included the question “Who are you doing this drug check for?” with options of: for self, to sell, for others, or skip. These categories are not mutually exclusive, and therefore, respondents were asked to check all that apply. When “for others” was selected, the service user was given the option to specify who the drug check was being performed for. All service users accessing drug checking in the time period were included in the analysis.

**Fig. 1:**
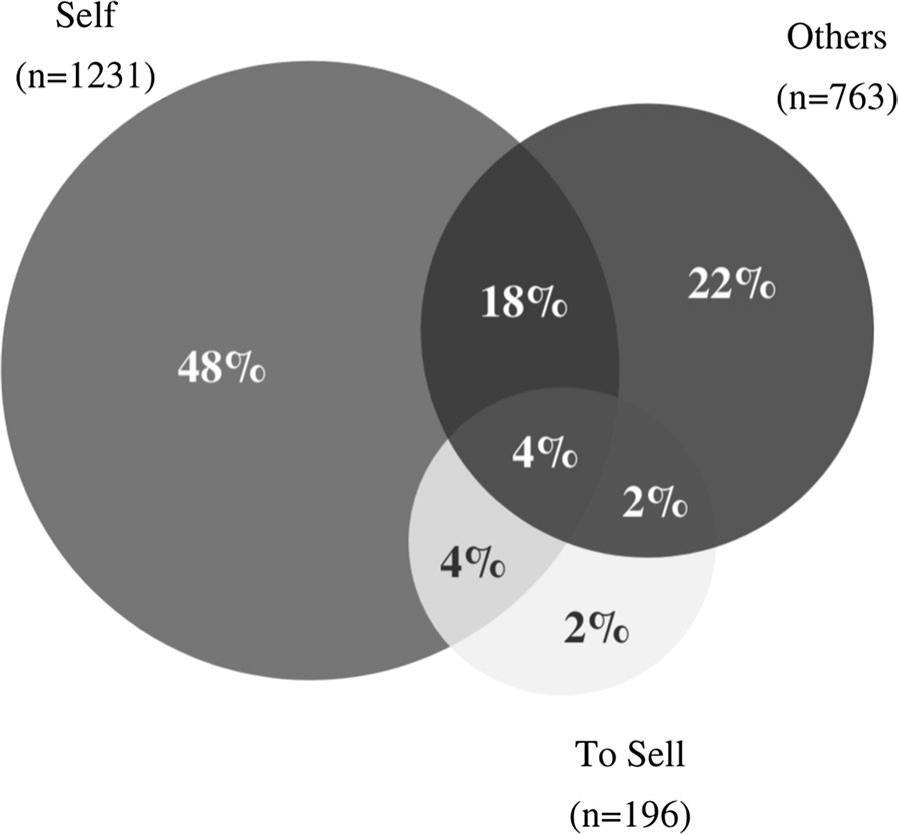
Distribution for the 1653 survey responses of service users who accessed a drug checking service and provided a response for who the drug check was being performed for. Responses could include either one or a combination of three groups: for self, for others, or to sell

To determine what portion of overall service users were checking for others, we first analyzed the distribution according to the three broad groups; self, others, and to sell. A Venn diagram was used to sort the complexities of overlap between the groups (Fig. 1).

Secondly, the open-ended answers to the “for others” specification were analyzed to code who was being included in these responses. Similar responses were grouped using keyword identifiers to establish broad categories of third party service use. These include friends (checking for friends, roommates, the community), family (spouse, parent, child, sibling, partner), outreach (support worker or community organization member which would include peers and outreach staff), suppliers (vendors, suppliers, dealers), and other (responses that did not fit into the other categories, as when an unknown substance was found). The number of service users who were checking for others, or in combination of for others with self or to sell, was then summarized by the frequency of responses for each third party category through a cross-tabulation analysis (Table 1).

## Results

Within the time period, 1991 people accessed the drug checking service. Of these, 1653 provided an answer to the survey question on the topic of checking for others, which is the subset used for the analysis of this study. Groupings were not mutually exclusive and included a large amount of overlap with 28% multiple responses (Fig. 1). Overall, 52% of the service users reported using the drug checking service for others or themselves and others. While 74% (1231) responded checking for self, 26% of those were also checking for others and other purposes. A total of 46% (763) answered checking for others and a total of 12% (196) confided in checking to sell.

Of the 763 total responses that were checking for others, 45% (345) specified who the other party was. The most common reason for checking for others was for friends, which comprised 52% of responses, and in most of these cases (65%), they reported to be checking for both themselves and their friends (Table 1). Thirty percent reported to be an outreach/support worker checking for others, where the majority were testing solely for others (87%) and a smaller portion were testing for both themselves and others (13%). When a service user reported checking drugs to sell to others, most often (69%) they listed these as friends. Our data show how family members are also accessing drug checking with 16% reporting checking for family members or themselves and their family, which included listing children, parents as well as partners.

**Table 1:** Cross-tabulation of the frequency of 345 service users who were checking for others, or in a combination of for others with self or to sell, that further specified who the third party was according to the general categories of friends, outreach, family, supplier or other

Specified other	Checking for others			Total	*N* (%)
	Other only	Other & Self	Other & To Sell		
Friends	57	116	18	179	52%
Outreach	91	11	7	104	30%
Family	38	17	1	56	16%
Suppliers	4	9	3	13	4%
Other	10	8	1	11	3%
Total	190	146	26		
*N* (%)	55%	42%	8%		

Multiple responses could be selected per service user; therefore, responses are not mutually exclusive and percentages do not add to 100%

## Discussion

Within a community drug checking service, we found that more than half (52%) of service users were checking drugs for reasons beyond individual use. This highlights that third parties are being used as a common approach to engage with a community drug checking service. These findings expand the current literature that demonstrates the utility of drug checking services as an intervention to mitigate individual risks within the harms of the unregulated market to the broader scale in the community setting.

Of those who specified who they were checking for, friends and family were mentioned most frequently (68%), pointing to the importance of drug checking as a relational practice. Due to stigma and criminalization, it is worth noting a possible positive bias to the response of checking for others, as people accessing the service may feel more comfortable admitting they are checking for a friend than themselves. Preliminary research has shown practices of care among friends to be seen in drug checking services [7], and we found this translated to the community setting as testing for friends represented 52% of the time people were checking for others. This demonstrates the care practices of third party checking and the social aspects of using substances, as is the possibility of having a designated person who checks substances for a group who may be using or buying together.

The inclusion of family members in supporting people who use drugs has been identified as increasing the reach of harm reduction outcomes [10]. Our findings show the supporting role that family members can play through drug checking. In most instances (68%), family members were checking for others only, highlighting how this service can provide a way for families to better understand substances and substance use to support their family members who are using drugs. It provides evidence for drug checking services being an access point to engage with harm reduction, enabling further openness in discussing substances and substance use. As community drug checking expands, we see benefits in engaging with families while also considering the challenges of consent and inherent power imbalances in families, such as a parent checking a child’s drugs unknowingly.

Outreach and service workers checking for others represented 30% of these responses. Currently, regulatory frameworks bar the transportation of substances for the purpose of drug checking by social service and healthcare workers. Therefore, this finding highlights that these workers who are accessing drug checking services on behalf of their clients are likely doing so despite a regulatory environment that prohibits it.

The outreach category also includes experiential workers and peer workers, as 12% of outreach workers reported checking not only for others, but themselves as well. Experiential and peer workers face disproportionate threats of criminalization and are further vulnerable to the impacts of enforcement [11]. Those providing outreach for drug checking are key in increasing the accessibility of a service to those who are less mobile within the city or those experiencing higher barriers to reach the service on their own behalf. As outreach workers represent a large portion of people accessing this service, regulation that enables social service workers to engage in drug checking services could further extend the reach of drug checking in addressing the harms of the current crisis.

Those accessing drug checking services for the purpose of selling or within the supply chain represented 12% of service users. There is likely under-reporting given the increased burden of criminalization on those who sell drugs, as this burden has been identified as a potential barrier to drug checking services for people who sell drugs [12]. Drug checking has been explored for its potential role in engaging people who sell drugs as a harm reduction practice with further reach to those vulnerable to unpredictability in the illicit supply [13–16]. Wallace et al. [9] highlight the potential of drug checking to act as a supply intervention and to potentiate market interventions by empowering consumers and providers with knowledge of the composition of their substances. Our findings confirm that indeed, drug checking services are used by people who sell drugs to provide some agency within the market and quality control for prospective consumers.

As a community-based response to overdose, drug checking benefits from considering and reaching the multiple publics impacted by substance use, stigma and overdose [17]. Furthermore, evaluations of drug checking as a public health intervention should incorporate measures of reach and impacts beyond the individual accessing services and individual behavioral change as the impacts of third party drug checking on reducing the risks and harms of illicit substances was not included in our data.

Overall, our work illustrates that drug checking services function as an intervention that extends well beyond the individual. Third party checking occurs frequently within drug checking and acts to increase accessibility to services as well as to engage the community in practices of care. This should inform drug checking services and policy makers to better facilitate the provision of and access to third party checking. Specifically, drug checking services should not be assumed to be a service limited to people who use drugs testing substances pre-consumption. Our findings suggest potential benefits for tailoring services to also reach and respond to others including family and friends, outreach and peer workers, and people selling drugs. Third parking checking will remain constrained without public health support and exemptions that recognize the challenges of drug checking within prohibition and substance use stigma.

## Data Availability

The data supporting the conclusions of this article are available on the Vancouver Island Drug Checking Project website, https://substance.uvic.ca/blog/tag/report/.
