# Correction to: Third party drug checking: accessing harm reduction services on the behalf of others

**DOI:** 10.1186/s12954-021-00548-7

**Published:** 2021-09-27

**Authors:** Ashley Larnder, Piotr Burek, Bruce Wallace, Dennis K. Hore

**Affiliations:** 1 Department of Chemistry, University of Victoria, Victoria, V8W 3V6 Canada; 2 Department of Computer Science, University of Victoria, Victoria, V8W 3P6 Canada; 3 School of Social Work, University of Victoria, Victoria, V8W 2Y2 Canada; 4 Canadian Institute for Substance Use Research, University of Victoria, Victoria, V8W 2Y2 Canada

## Correction to: Harm Reduct J (2021) 18:99 https://doi.org/10.1186/s12954-021-00545-w

After publication of the original article [1], an error was identified in the last sentence.

The sentence currently reads: Third parking checking will remain constrained without public health support and exemptions that recognize the challenges of drug checking within prohibition and substance use stigma.

The sentence should read: Third party checking will remain constrained without public health support and exemptions that recognize the challenges of drug checking within prohibition and substance use stigma.

The original article has been corrected.

## References

[1] Larnder A (2021). Harm Reduct J.

